# The Reliability of Three-Dimensional Landmark-Based Craniomaxillofacial and Airway Cephalometric Analysis

**DOI:** 10.3390/diagnostics13142360

**Published:** 2023-07-13

**Authors:** Kan Yao, Yilun Xie, Liang Xia, Silong Wei, Wenwen Yu, Guofang Shen

**Affiliations:** 1 Department of Oral and Cranio-Maxillofacial Surgery, Shanghai Ninth People’s Hospital, Shanghai Jiao Tong University School of Medicine, Shanghai 200011, China; 117080@sh9hospital.org.cn (K.Y.); 119026@sh9hospital.org.cn (L.X.);; 2 College of Stomatology, Shanghai Jiao Tong University, Shanghai 200011, China; 3 National Center for Stomatology, National Clinical Research Center for Oral Diseases, Shanghai Key Laboratory of Stomatology, Shanghai Research Institute of Stomatology, Shanghai 200011, China; 4 Department of Stomatology, Ren Ji Hospital, Shanghai Jiao Tong University School of Medicine, Shanghai 200127, China; xieyilun@renji.com

**Keywords:** cephalometric analysis, three-dimensional, CT, cranio–maxillofacial related disorders, airway

## Abstract

Cephalometric analysis is a standard diagnostic tool in orthodontics and craniofacial surgery. Today, as conventional 2D cephalometry is limited and susceptible to analysis bias, a more reliable and user-friendly three-dimensional system that includes hard tissue, soft tissue, and airways is demanded in clinical practice. We launched our study to develop such a system based on CT data and landmarks. This study aims to determine whether the data labeled through our process is highly qualified and whether the soft tissue and airway data derived from CT scans are reliable. We enrolled 15 patients (seven males, eight females, 26.47 ± 3.44 years old) diagnosed with either non-syndromic dento–maxillofacial deformities or OSDB in this study to evaluate the intra- and inter-examiner reliability of our system. A total of 126 landmarks were adopted and divided into five sets by region: 28 cranial points, 25 mandibular points, 20 teeth points, 48 soft tissue points, and 6 airway points. All the landmarks were labeled by two experienced clinical practitioners, either of whom had labeled all the data twice at least one month apart. Furthermore, 78 parameters of three sets were calculated in this study: 42 skeletal parameters (23 angular and 19 linear), 27 soft tissue parameters (9 angular and 18 linear), and 9 upper airway parameters (2 linear, 4 areal, and 3 voluminal). Intraclass correlation coefficient (ICC) was used to evaluate the inter-examiner and intra-examiner reliability of landmark coordinate values and measurement parameters. The overwhelming majority of the landmarks showed excellent intra- and inter-examiner reliability. For skeletal parameters, angular parameters indicated better reliability, while linear parameters performed better for soft tissue parameters. The intra- and inter-examiner ICCs of airway parameters referred to excellent reliability. In summary, the data labeled through our process are qualified, and the soft tissue and airway data derived from CT scans are reliable. Landmarks that are not commonly used in clinical practice may require additional attention while labeling as they are prone to poor reliability. Measurement parameters with values close to 0 tend to have low reliability. We believe this three-dimensional cephalometric system would reach clinical application.

## 1. Introduction

Cephalometric analysis, first introduced by Hofrath H [1] and Broadbent BH [2], has been a standard diagnostic tool in orthodontics and craniofacial surgery for the last few decades [3,4,5,6]. As is well known in orthodontic and orthognathic surgery fields, the accurate quantification of deformities and precise surgical planning requires the digitization (detection and localization) of cranio–maxillofacial (CMF) landmarks. Initially, cephalometry was focused on skeletal structures and could only assess two dimensions [7,8,9,10,11]. As two-dimensional cephalometric analysis evolved, soft-tissue cephalometric analyses were established for the evaluation of attendant soft-tissue changes and esthetic considerations [12,13,14,15,16]. Moreover, as the factor that patients with obstructive-sleep disordered breathing (OSDB) show certain craniofacial defects that may influence pharyngeal patency received attention, cephalometric analyses focused on airways were introduced [17,18,19]. Two-dimensional cephalometry is widely adopted in clinical practice due to its simplicity, convenience, and certain reliability. However, conventional cephalometry is susceptible to analysis bias due to the difficulty in determining some landmarks with high accuracy and reliability because of the superimposition of anatomic structures [20,21,22]. 

To overcome the drawback, three-dimensional cephalometric analysis was introduced. The fundamental basis for digital three-dimensional cephalometry is the data on the head and facial structure. Currently, many technologies (such as computed tomography (CT) [23,24], cone beam computed tomography (CBCT) [25], magnetic resonance imaging (MRI) [26,27], and facial scanning [28]) can provide high-resolution images without overlapping or distortion, which results in high-quality diagnostic images. In three-dimensional cephalometry, more landmarks, reference planes, and measurement parameters can be selected to enrich the analysis content of bone, soft tissue, and airway anatomy [29,30,31,32,33,34,35,36]. Measuring volumes (especially airway volumes) and visual asymmetry evaluation become possible. Three-dimensional data provide potentially useful information compared to two-dimensional data. However, this also brings a large amount of redundant information, which poses high demands on the processing of three-dimensional data. In clinical practice, landmark digitization (especially three-dimensional) is still performed manually, which is time-consuming, error-prone, and experience-dependent. Fast and reliable automated landmark digitization systems are highly desirable by clinicians. Recently, many automated landmark digitization systems have been established with a certain level of accuracy, motivated by the successes of machine learning in the field of medical image analysis [37,38,39,40,41]. 

To create a more reliable and user-friendly three-dimensional cephalometric system, we conducted a study to establish an automated multimodal measurement system that includes hard tissue, soft tissue, and airways. The first issue that needs to be addressed is high-quality training data. The performance of machine learning models is based on training data. Since it is difficult to surpass training data, unreliable data is difficult to train robust models. The lack of high-quality data is one of the obstacles to improving the accuracy of machine learning, especially in the field of medical image analysis [42,43,44,45]. Although many studies have been conducted on the reliability of landmarks [46,47,48], most are based on commonly used landmarks in clinical practice. Our system hopes to explore more clinical information and has introduced some non-commonly used landmarks, such as the ones used to evaluate soft tissue nasal anatomy. For these landmarks that most clinical experts have not marked, their reliability remains questionable. In addition, the former studies also indicate that important variations were observed in the experimental methods regarding the parameters of image acquisition, software, types of visualization, and the marked anatomic references. After careful consideration, CT data were chosen as the data source for our measurement system. Most patients with cranio–maxillofacial related disorders need to undergo a CT scan for diagnosis and surgical design. Soft tissue and airway structures can be obtained from CT data as well, thus avoiding inconvenience and extra radiation. To ensure the accuracy of our training data as much as possible, we must perform a reliability check on the landmarks and measurement parameters we select. At the same time, we also want to evaluate whether clinical experts can achieve a high level of reliability based on the definition of some less commonly used landmarks. We believe this is a necessary step before establishing a reliable automatic three-dimensional measurement system. This will make the thousands of labeled data we establish based on our process more convincing. We also plan to open-source the data in the future and promote the application of three-dimensional craniofacial measurement systems in clinical practice. We hope that our system can bring more efficient and detailed clinical data to clinicians and patients in the future. 

## 2. Materials and Methods

CT scans for this study were derived from a pre-existing clinical database of pre-orthognathic treatment records, and the study protocol was approved by the institution review board of Shanghai Ninth People’s Hospital, Shanghai Jiao Tong University School of Medicine (SH9H-2022-T45-2). No additional radiographic images were taken for study purposes. All the CT scans were taken in 2020 and anonymized. Data from 15 patients (seven males, eight females, 26.47 ± 3.44 years old) diagnosed with either nonsyndromic dento–maxillofacial deformities or OSDB were included in this study. The CT DICOM files [49] were converted into point cloud data with voxel size of 0.5 × 0.5 × 0.5 mm [3], the scalar of which is the value of Hounsfield unit (Hu) [50]. The data were resampled using cubic spline interpolation. This method has been shown to be effective for resampling data due to its good interpolating performance and processing efficiency in previous studies [51,52,53,54]. SciPy package version 1.7.3 [55] based on Python version 3.7.3 [56] was used for data resampling. 

**Figure 1:**
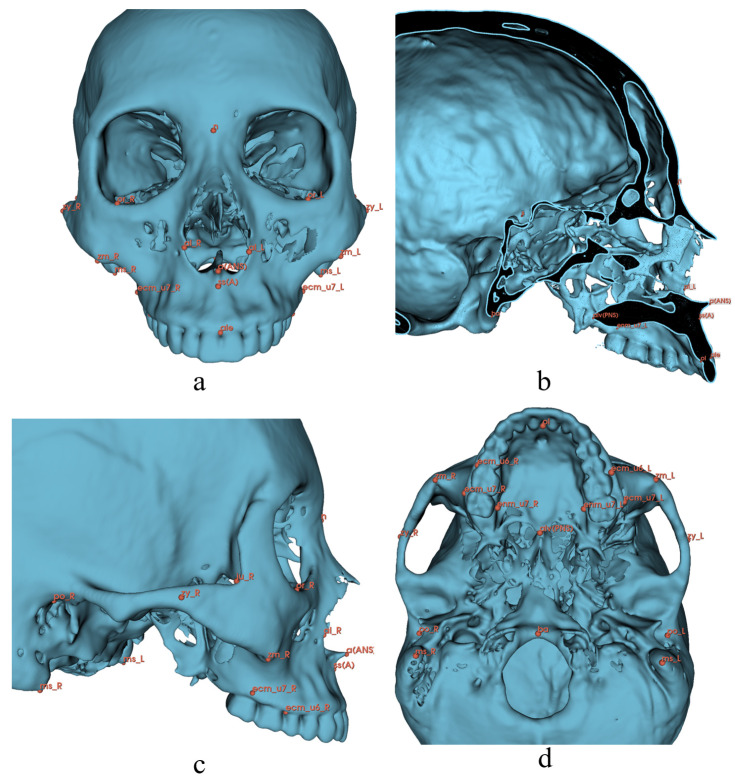
Cranial landmarks for 3D analysis. Definitions are provided in Table 1. (**a**) Norma Frontalis; (**b**) Norma Medialis; (**c**) Norma Lateralis; (**d**) Norma Basalis.

**Figure 2:**
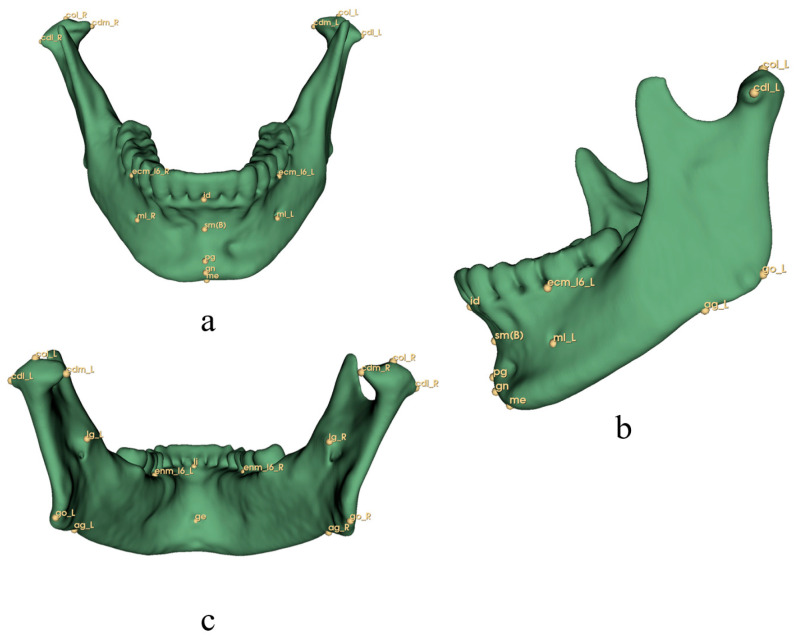
Mandibular landmarks for 3D analysis. Definitions are provided in Table 1. (**a**) Norma Frontalis; (**b**) Norma Lateralis; (**c**) Norma Occipitalis.

**Figure 3:**
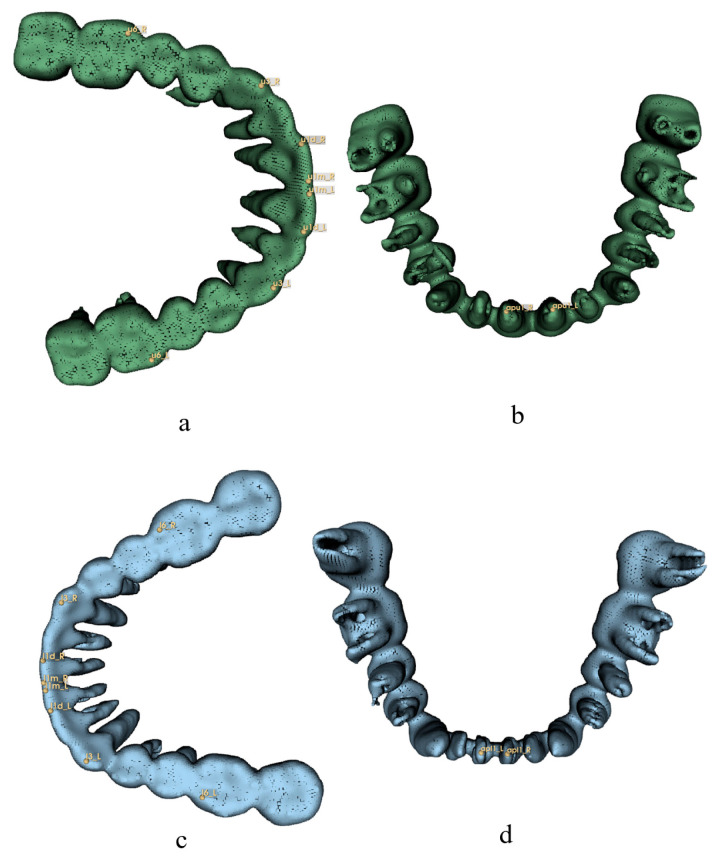
Teeth landmarks for 3D analysis. Definitions are provided in Table 1. (**a**) Norma Basalis, upper teeth; (**b**) Norma Verticalis, upper teeth; (**c**) Norma Verticalis, lower teeth; (**d**) Norma Basalis, lower teeth.

**Figure 4:**
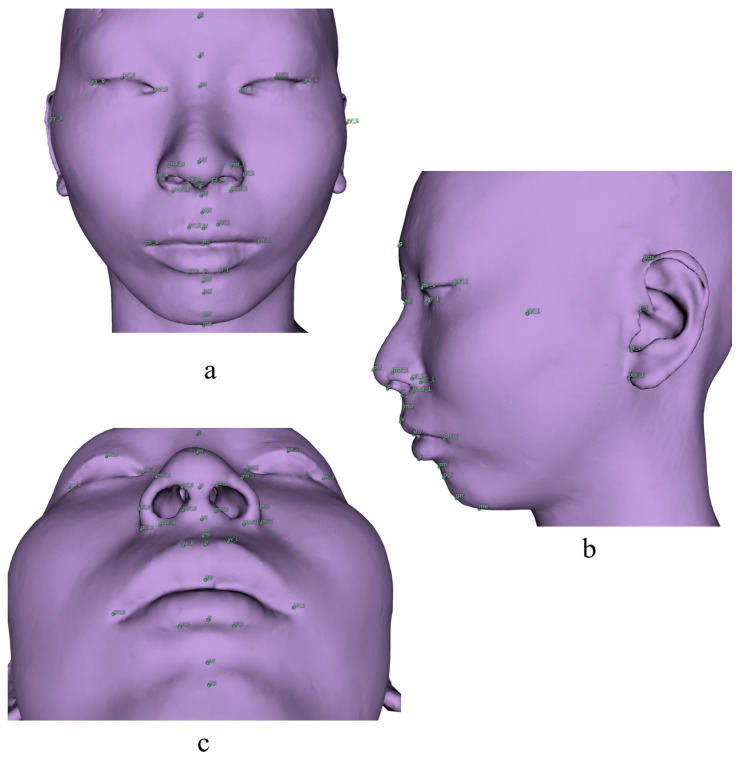
Soft tissue landmarks for 3D analysis. Definitions are provided in Table 1. (**a**) Norma Frontalis; (**b**) Norma Lateralis; (**c**) Norma Basalis.

**Figure 5:**
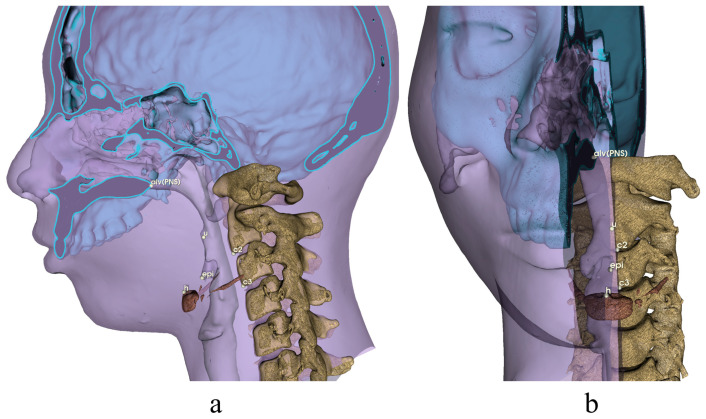
Airway landmarks for 3D analysis. Definitions are provided in Table 1. (**a**) Norma Medialis; (**b**) Norma Frontalis.

**Figure 6:**
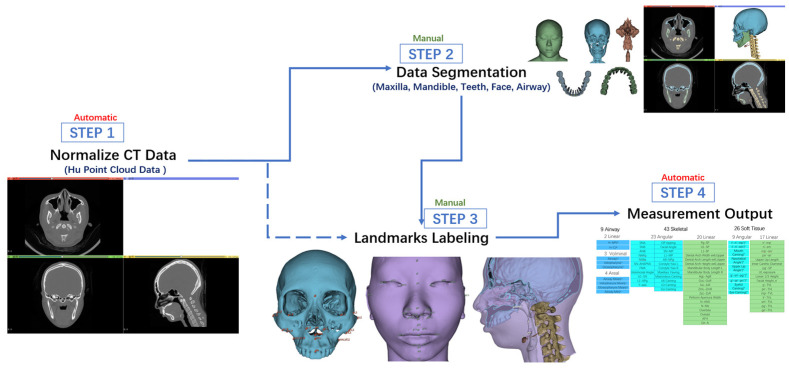
Schematic diagram of our semi-automatic system in this study.

In this study, 126 landmarks were adopted and were divided into five sets by region: 28 cranial points (8 median and 10 bilateral), 25 mandibular points (7 median and 9 bilateral), 20 teeth points (10 bilateral), 48 soft tissue points (14 median and 17 bilateral), and 6 airway points (6 median). The alv(PNS) point was included in both cranial and airway sets (Table 1, Figure 1, Figure 2, Figure 3, Figure 4 and Figure 5) [3,33,34,57,58,59]. All the landmarks were labeled by two experienced clinical practitioners using 3D Slicer software (version 4.13.0, https://www.slicer.org/ accessed on 23 March 2022.) [60], either of whom had labeled all the data twice at least one month apart. Three-dimensional reconstructions of the skeletal structure, teeth, soft tissue, and upper airway were created and exported as VTK [61] files using 3D Slicer before labeling (Figure 6).

Based on landmarks, seven reference planes were created: Frankfort horizontal plane (FH), Sagittal plane (SP), Horizontal plane (HP), Coronal plane (CP), Mandibular plane (MP), Occlusal plane (OP), and True vertical line plane (TVL) (Table 2). Furthermore, 78 parameters of three sets were used in this study: 42 skeletal parameters (23 angular and 19 linear), 27 soft tissue parameters (9 angular and 18 linear), and 9 upper airway parameters (2 linear, 4 areal, and 3 voluminal) (Table 3).

The reliability consists of two aspects: inter-examiner reliability and intra-examiner reliability [63]. Inter-examiner reliability refers to the consistency between different examiners while intra-examiner reliability means the ability of an examiner to record the same conditions the same way over time. In this study, intraclass correlation coefficient (ICC) was used to evaluate inter-examiner and intra-examiner reliability [6,64]. For inter-examiner reliability, the values from two sets of landmark coordinates and cephalometric analyses were used, and ICC estimates and their 95% confident intervals were calculated based on a single-measurement, absolute-agreement, and two-way random-effects model. For intra-examiner reliability, the average value of two sets of landmark coordinate values and cephalometric analyses from each examiner was used. ICC values less than 0.5 are indicative of poor reliability, values between 0.5 and 0.75 indicate moderate reliability, values between 0.75 and 0.9 indicate good reliability, and values greater than 0.90 indicate excellent reliability. All the ICC estimates were calculated using Pingouin statistical package version 0.5.2 [65] based on Python version 3.7.3 [56]. 

## 3. Results

### 3.1. The Intra- and Inter-Examiner Reliability of Landmark Coordinate Values

We first compared the intra- and inter-examiner ICCs for all landmark coordinate values with 15 samples together. Each landmark is a point of R^3^ in the left–posterior–superior coordinate system (LPS). The inter-examiner and the intra-examiner ICCs for each landmark are listed in Table 4. The overwhelming majority of the landmarks showed excellent intra- and inter-examiner reliability. The intra-examiner reliability of landmarks is better than the inter-examiner reliability. For poorly performing landmarks, there are two conditions. (1) The ICC value is poor (less than 0.75) in the reproducibility in the S direction of pg’; the reproducibility and repeatability in the P direction of zy’_L; the reproducibility in the S direction of zy’_R; and the repeatability in the P direction. (2). The ICC value is good but the lower bound of 95% confidence interval is less than 0.50, which might indicate potential poor performance and is only observed in the reproducibility in the L direction of or_L, ecm_u6_L, ecm_l6_L, ecm_l6_R, u1d_R, mc’_L, sbal’_R, and vi’_L; the P direction of zy_L, gn, me, ag_R, ecm_l6_L, pg’, gn’, al’_L, and sbal’_R; the S direction of ecm_u7_L, g’, se’, pn’, sn’, gn’, sbal’_R, vs’_L, vs’_R, zy’_L, and zy’_R; the LS direction of ecm_u6_R, ecm_u7_R, enm_u7_L, and enm_u7_R; and the LPS direction of sbal’_R. The majority of the landmarks with poor performance are non-commonly used ones. 

### 3.2. The Intra- and Inter-Examiner Reliability of Measurement Parameters

The intra- and inter-examiner ICCs for measurement parameters with 15 samples were then calculated. The inter-examiner and the intra-examiner ICCs for each parameter are listed in Table 5. Most of the parameters showed excellent intra- and inter-examiner reliability. For skeletal parameters, angular parameters indicated better reliability, while linear parameters performed better for soft tissue parameters. The intra- and inter-examiner ICCs of airway parameters referred to excellent reliability. For poorly performing parameters, two conditions were also observed: (1) The ICC value is poor (less than 0.75) in the reproducibility and repeatability of Maxillary Yawing|° and the repeatability of Go Canting|°, mp’-sm’|^d^, pn’-sn’|^d^, Inner Canthic Diameter|^d^, and Upper Vermilion Width|^d^. (2) The ICC value is good, but the lower bound of 95% confidence interval is less than 0.50 in the reproducibility of Upper Vermilion Width|^d^ and the repeatability of Condyle Yaw R|°. The repeatability of examiner 2 seemed to be relatively poor.

## 4. Discussion

In this study, we evaluated the inter- and intra-examiner reliability of our three-dimensional landmark-based cranio–maxillofacial and airway cephalometric analysis in both landmark and measurement parameter levels. We aimed to determine whether the data labeled through our process are highly qualified and whether the soft tissue and airway data derived from CT scans are reliable.

Landmarks in our study were R^3^ points, based on which all the measurement parameters were calculated. As a result, the reliability of landmarks matters. Bookstein introduced three types of landmarks (I, II, and III) to elucidate their character and degree of reliability [66]. To reach a more robust measurement system, the points derived from anatomical structures (Bookstein class I) were preferred [66]. Our study showed that the reliability of most of the landmarks was excellent, though we still found some with relatively poor performance. Landmarks that are not commonly used in clinical practice may require additional attention as they may have poor reliability. We will take extra care when labeling these landmarks. 

For the skeletal cranial landmarks, the poorest performing one was the anterior–posterior direction of the Zygion (zy), which is defined as the most lateral point of the zygomatic arch. This point is not based on specific anatomical structure and requires the examiner to estimate the position based on visual observation. Due to the arched shape of the zygomatic arch, small fluctuations in the left–right direction can cause significant changes in the anterior–posterior direction. Currently, the Zygion (zy) is mainly used to measure the width of the face, so as long as the left–right direction fluctuation is small, it has little impact on the measurement output. However, for safety reasons, we also selected some alternative landmarks: the Mastoidale (ms), the Zygomaxillare (zm), and the Jugale (ju), which had much better repeatability and reproducibility. 

While the landmarks belonging to mandibular, teeth, and airway structure showed great repeatability and reproducibility in our study, some points of soft tissue were suboptimal. Like the skeletal zygion (zy), both the reproducibility and repeatability of the anterior–posterior direction of the soft tissue zygion (zy’) were poor. Unlike skeletal pogonion (pg), we noticed the low inter-observer ICC of the inferior–superior direction of the soft tissue pogonion (pg’), which indicated the potential labeling deviation between the two raters. After reviewing our data, we speculated that the possible reason was the discrepancy between the curvature of soft tissue and hard tissue in facial contour analysis. The soft tissue was more flexible and had lower radii of curvature than hard tissue, which made it harder to locate the pg’.

To improve the reliability of the landmarks with relatively poor performance, we initiated a project to optimize the Bookstein type II landmark labeling with computer assistance. The program would relocate the labeled landmark based on numerical calculation. Just like other researchers [37,38,39,40,67,68], we attempted to establish an automatic labeling system based on a machine-learning technique as well.

In our measurement system, the output parameters were calculated by landmarks and rules. The reliability of measurement indicators might not be completely equivalent to the reliability of landmarks. Thus, we evaluated the repeatability and reproducibility of parameters as well. In our study, linear parameters of hard tissue seemed to be more robust than the angular ones, while the opposite is true for parameters of soft tissue. Maxillary Yawing|° demonstrated poor reproducibility and repeatability, while Go Canting|° showed low repeatability. Maxillary Yawing|° is designed to evaluate the yawing of the maxilla, the closer the value of which is to 0, the less skewed maxilla an individual has. Most patients have low maxillary yawing values, so the value of Maxillary Yawing|° is close to 0, and we believe this is the main reason for its low reliability. Go Canting|° is derived from the go (Gonion) points. The consistency of examiner 2’s Go Canting|° in our study was relatively poor, while its go points consistency was still at a relatively high level. This indicated that there may be an amplification of deviations in the calculation process from point to measurement value. We found similar phenomena in Upper Vermilion Width|^d^ and Inner Canthic Diameter|^d^. As a result, in our subsequent studies, we will explore and quantify the changes in errors between points and measurement values. 

In our study, the parameters of the airway were stable. For airway indicators, a three-dimensional measurement may describe the airway in a better way. Since OSDB is caused by upper airway collapse, the aim of clinical treatment for OSDB is to find and relieve the narrowest region of the airway [69,70,71,72,73,74,75,76,77,78,79,80]. Fortunately, the important indicator of the narrowest area of the airway (Airway, Min|^a^ in our study) is proven to be robust.

**Figure 7:**
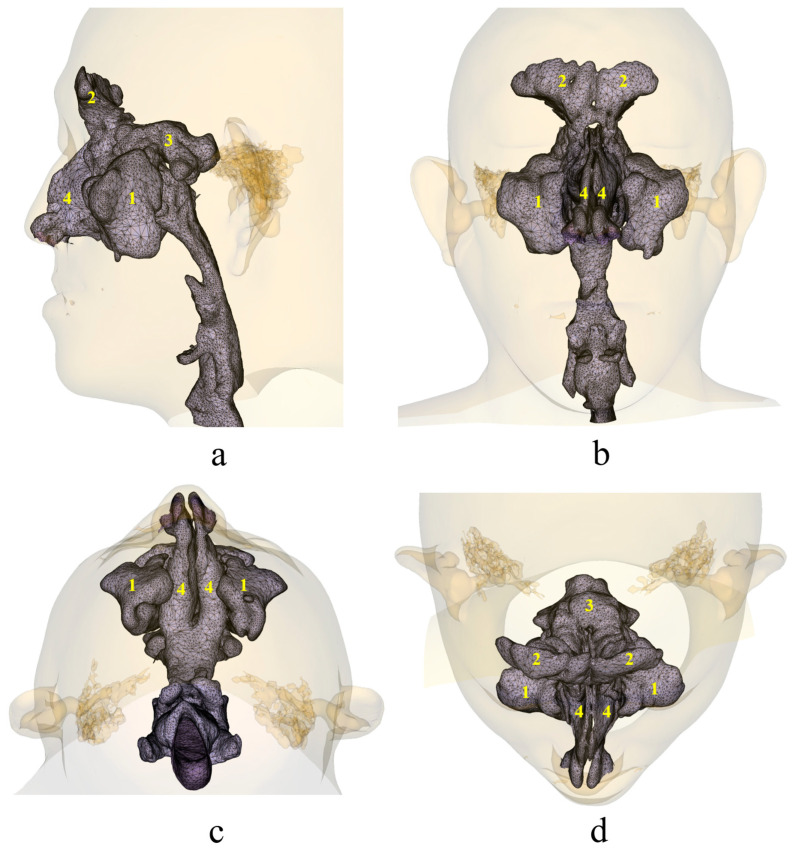
Images of nasal cavity reconstruction: (**a**) Norma Lateralis, (**b**) Norma Frontalis, (**c**) Norma Basalis, (**d**) Norma Verticalis. In images above, (**1**) represents the maxillary sinus air cavity structure, (**2**) represents the frontal sinus air cavity structure, (**3**) represents the ethmoid sinus air cavity structure, and (**4**) represents the nasal cavity airway structure.

Unfortunately, only nine parameters of the airway were adopted in this study. One reason is that research studies on airway morphology are still in their very early stages, and there are relatively few parameters that are clinically applicable. On the other hand, due to technical limitations, some indicators are too complex to be calculated efficiently and stably. For example, in airway assessment, nasal cavity volume is actually a very important parameter since nasal stenosis can also lead to OSDB. Currently, the segmentation of the nasal cavity is still based on air/soft tissue thresholds. Since the nasal cavity is not only connected to the pharynx but also to the nasal sinuses, segmentation based on thresholds will usually segment out the nasal sinus cavity (Figure 7). To exclude the nasal sinus cavity, manual erasure is required, which is labor-intensive and may lead to decreased accuracy due to unclear boundaries between the nasal sinuses and the nasal cavity. In addition, as the size of the nasal sinuses varies among patients, including them in nasal cavity volume measurement could not indicate nasal cavity morphology correctly. As the measurement of the nasal cavity should be based on the efficient and accurate segmentation of the relevant structure of the nasal cavity, which is currently beyond the scope of this study, this part of the study did not include airway measurements related to the nasal cavity. We plan to further explore this in subsequent studies.

The work in this paper is the predecessor work of our automatic 3D cephalometric system project as well. Due to the large amount of information added by 3D measurement compared to 2D measurement, the high cost of manual processing has become a major obstacle for 3D measurement systems to move towards clinical application. We believe that an automatic processing system is an effective solution. We plan to use machine-learning techniques to achieve automatic labeling of landmarks. To achieve excellent auto-labeling models, data with correct labeling need to be prepared first. Our work proved that the reliability of the system mentioned in this paper was excellent; thus, we believe the training data could be highly qualified. Talking about the 3D cephalometric system, whether a landmark-based cephalometric technique is still applicable is a question worth pondering. Compared to 2D parameters, we could have parameters of symmetry, volume, and so on, and for these indicators, the role of landmarks may be the key to quickly locating the region of interest. As a result, our 3D system is designed to be able to keep updating our landmark list automatically to adapt to new demands.

## 5. Conclusions

In summary, we introduced a three-dimensional measurement system, the content of which covers hard tissue, soft tissue, and the airway. The repeatability and reproducibility of the measurement system were evaluated and proven to be robust enough for clinical practice by two aspects: landmark coordinates and measurement parameters. The data labeled through our process are qualified, and the soft tissue and airway data derived from CT scans are reliable. Landmarks that are not commonly used in clinical practice may require additional attention while labeling as they are prone to poor reliability. Measurement parameters with values close to 0 tend to have low reliability. The role of landmarks may be key to quickly locating regions of interest in successor three-dimensional cephalometric systems. We believe this three-dimensional cephalometric system would reach clinical application and help clinical practitioners improve the quality of clinical practice.

## Figures and Tables

**Table 1:** Definitions and abbreviations of the landmarks. Non-commonly used landmarks are marked in red.

Classification		3D Notation	Landmark	Definition
Cranial	Median	s	Sella	The central point of the sella turcica
		ba	Basion	The most inferior median point on the foramen magnum’s anterior rim
		n	Nasion	Intersection of the nasofrontal sutures in the median plane
		a(ANS)	Acanthion (ANS)	Most anterior tip of the anterior nasal spine; also known as anterior nasal spine (ANS)
		ss(A)	Subspinale	The deepest point seen in the profile view below the anterior nasal spine; also known as orthodontic point A
		alv(PNS) *	Alveolon (PNS)	Median point, at the rear of the hard palate, of a line joining the posteriormost alveolar margins; also known as posterior nasal spine (PNS)
		ale	Alveolare	Median point at the inferior tip of the bony septum between the upper central incisors
		ol	Orale	Median most inferior point of the maxillary symphysis; on the lingual surface
	Bilateral	po	Porion	Most superior point on the upper margin of the external auditory meatus
		ms	Mastoidale	The inferiormost projecting point of the tip of the mastoid process
		or	Orbitale	Most inferior point on the inferior orbital rim; usually falls along the lateral half of the orbital margin
		al	Alare	Instrumentally determined as the most lateral point on the nasal aperture in a transverse plane
		zm	Zygomaxillare	Most inferior point on the zygomaticomaxillary suture
		ju	Jugale	Vertex of the posterior zygomatic angle; between the vertical edge and horizontal part of the zygomatic arch
		zy	Zygion	Instrumentally determined as the most lateral point on the zygomatic arch
		ecm_u6	Ectomolare U6	Most lateral point on the buccal alveolar margin; at the mesial buccal edge of the first molar position
		ecm_u7	Ectomolare	Most lateral point on the buccal alveolar margin; at the center of the second molar position
		enm_u7	Ectomolare	Most lateral point on the lingual alveolar margin; at the center of the second molar position
Mandibular	Median	id	Infradentale	Median point at the superior tip of the septum between the mandibular central incisors
		li	Linguale	Median most superior point of the mandibular symphysis; on the lingual surface
		sm(B)	Supramentale	Deepest median point in the groove superior to the mental eminence; also known as orthodontic point B
		pg	Pogonion	Most anterior median point on the mental eminence of the mandible
		gn	Gnathion	Median point halfway between pg and me
		me	Menton	Most inferior median point of the mental symphysis (may not be the inferior point on the mandible as the chin is often clefted on the inferior margin)
		ge	Genion	Most projecting tip of the internal mental spine on the lingual surface of the mandible
	Bilateral	col	Condyle	The most superior point of the mandibular condyle
		cdl	Condylion laterale	Most lateral point on the mandibular condyle
		cdm	Condylion mediale	Most medial point on the mandibular condyle
		go	Gonion	Point on the rounded margin of the angle of the mandible, bisecting two lines, one following vertical margin of ramus and one following horizontal margin of corpus of mandible
		ag	Antegonion	Apex of the antegonial notch
		ecm_l6	Ectomolare L6	Buccal alveolar margin; at the mesial buccal edge of the lower first molar position
		ml	Mentale	Most inferior point on the margin of the mandibular mental foramen
		lg	Lingulare	Superiormost point of the lingula of the mandible
		enm_l6	Endomolare L6	Lingual alveolar margin; at the mesial buccal edge of the lower first molar position.
Teeth	Bilateral	u1d	Upper incisor distal	The distal point of the incisal edge of the upper central incisor
		u1m	Upper incisor mesial	The mesial point of the incisal edge of the upper central incisor
		apu1	Apex of U1	The apex of the upper central incisor
		u3	U3	The cusp tip of upper canine
		u6	U6	The mesiobuccal cusp tip of the upper first molar
		l1d	Lower incisor distal	The distal point of the incisal edge of the lower central incisor
		l1m	Lower incisor mesial	The mesial point of the incisal edge of the lower central incisor
		apl1	Apex of L1	The apex of the lower central incisor
		l3	L3	The cusp tip of lower canine
		l6	L6	The mesiobuccal cusp tip of the lower first molar
Soft Tissue	Median	g’	Glabella	Most anterior midline point on the forehead; in the region of the superciliary ridges
		n’	Nasion	Point directly anterior to the nasofrontal suture, in the midline, overlying n
		se’	Sellion	Deepest midline point of the nasofronal angle
		pn’	Pronasale	The most anteriorly protruded point of the apex nasi; in the case of a bifid nose, the more protruding tip is chosen
		sn’	Subnasale	Median point at the junction between the lower border of the nasal septum and the philtrum area
		c’	Columella	Midpoint of the nasal columella crest intersecting a line between the two cs′ points
		ls’	Labiale superius	Midpoint of the vermilion border of the upper lip
		mp ’	Mid-philtrum	Point midway between sn′ and ls′; in the median plane
		sto’	Stomion	Midline point of the labial fissure when the lips are closed naturally with teeth shut in the natural position
		li’	Labiale inferius	Midpoint of the vermilion border of the lower lip
		sm’	Supramentale	Deepest midline point of the mentolabial sulcus
		me’	Menton	Most inferior median point of the chin
		pg’	Pogonion	Most anterior midpoint of the chin; located on the skin surface anterior to the identical bony landmark of the mandible
		gn’	Gnathion	Median point halfway between pg′ and me′
	Bilateral	al’	Alare	The most lateral point on the nasal ala
		ma’	Mid-alare	Midpoint on the nasal alar where the ala thickness (not width) is measured
		ac’	Alar curvature point	The most posterolateral point of the curvature of the base line of each nasal ala
		sbal ’	Subalare	Most inferior point of the earlobe
		mc’	Mid-columella	Midpoint of the nasal columella crest on either side where the columella thickness is measured
		cs’	Columella superius	Most superior point on each columella crest of the nose; level with the top of the corresponding nostril
		vs’	Vermilion superius	Most superior point of the vermilion border of the upper lip at its apex on either side
		ch’	Cheilion	Outer corners of the mouth where the outer edges of the upper and lower vermilions meet
		vi’	Vermilion inferius	Most inferolateral point of the vermilion border of the lower lip at the maximum curve change on either side
		obs’	Otobasion superius	Most superior point of attachment of the ear helix to the temporal region of the head
		t’	Tragion	Located at the notch above the tragus of the ear (the cartilaginous projection anterior to the external auditory canal) where the upper edge of the cartilage disappears into the skin of the face
		it’	Intertragion	Apex of groove between the tragus and antitragus
		obi’	Otobasion inferius	Most inferior point of attachment of the ear lobe with the cheek
		ps’	Palpebrale superius	Most superior point on the margin of the upper eyelid
		en’	Endocanthion	Most medial point of the palpebral fissure; at the inner commissure of the eye; best seen when subject is gazing upward
		ex’	Exocanthion	Most lateral point of the palpebral fissure; at the outer commissure of the eye; best seen when subject is gazing upward
		zy’	Zygion	Most lateral point overlying each zygomatic arch; identified as the point of maximum bizygomatic breadth of the face
Airway	Median	alv(PNS) *	Alveolon(PNS)	Median point, at the rear of the hard palate, of a line joining the posterior most alveolar margins; also known as posterior nasal spine (PNS)
		u	Uvula	The tip of uvula
		h	Hyoid	The superior anterior median point of hyoid
		c3	C3 anterius inferius	The inferior anterior median point of the third cervix
		c2	C2 anterius inferius	The inferior anterior median point of the second cervix
		epi	Epiglottis	Most inferior point of epiglottic vallecula

* The alv(PNS) point is shared by either cranial or airway structure.

**Table 2:** Definitions and abbreviations of the planes.

Notation	Plane	Definition
FH	Frankfort horizontal plane	Plane fitted by orbitales and porions of both sides *
SP	Sagittal plane	Plane passing through the nasion and basion perpendicular to the FH plane
HP	Horizontal plane	Plane passing through the nasion parallel to the FH plane
CP	Coronal plane	Plane passing through the nasion perpendicular to the FH and SP plane
MP	Mandibular plane	Plane passing through the gnathion and gonions of both sides
OP	Occlusal plane	Plane passing through u1d, u1m, and u6 of both sides and the midpoint of left and right
TVL	True vertical line plane	Plane passing through the sn’ perpendicular to the FH and SP plane

* Plane was fitted by ordinary least squares regression [62].

**Table 3:** Definitions and abbreviations of the measurement parameters.

Classification		Measurement Parameters	Definition
Skeletal	Angular	SNA|° ^1^	s-n-ss(A) angle; projected on SP
		SNB|°	s-n-sm(B) angle; projected on SP
		ANB|°	ss(A)-n-sm(B) angle; projected on SP
		NAPg|°	n-ss(A)-pg angle; projected on SP
		NSBa|°	n-s-ba angle; projected on SP
		SN-ANSPNS|°	Angle between s-n and a(ANS)-alv(PNS); projected on SP
		FMA|°	Angle between FH and MP; projected on SP
		Interincisal Angle|°	Angle between the axes of the upper and lower central incisors (average); projected on SP ^2^
		AB-NPg|°	Angle between ss(A)-sm(B) and n-pg; projected on SP
		U1-SN|°	Angle between s-n and the axes of the upper central incisors (average); projected on SP ^2^
		L1-APg|°	Angle between ss(A)-pg and the axes of the lower central incisors (average); projected on SP ^2^
		Maxillary Yawing|°	Angle between a(ANS)-alv(PNS) and SP; projected on HP
		Mastoideus Canting|°	Angle between HP and the line connecting bilateral ms points; projected on CP
		U6 Canting|°	Angle between HP and the line connecting bilateral u6 points; projected on CP
		U3 Canting|°	Angle between HP and the line connecting bilateral u3 points; projected on CP
		Go Canting|°	Angle between HP and the line connecting bilateral go points; projected on CP
		Y-axis|°	Angle between gn-s and FH; projected on SP
		OP Tipping|°	Angle between OP and HP; projected on SP
		Facial Angle|°	Angle between n-pg and FH; projected on SP
		SN-MP|°	Angle between s-n and MP; projected on SP
		L1-MP|°	Angle between MP and the axes of the lower central incisors (average); projected on SP ^2^
		Condyle Yaw L|°	Angle between left cdl-cdm and SP; projected on HP
		Condyle Yaw R|°	Angle between right cdl-cdm and SP; projected on HP
	Linear	Pg-SP|^d 3^	Distance from pg to SP
		U1-SP|^d^	Distance from u1 to SP ^2^
		L1-SP|^d^	Distance from l1 point to SP ^2^
		Dental Arch Width ex6, Upper|^d^	Distance between left and right ecm_u6 points
		Dental Arch Length ex6, Upper|^d^	Distance from ale to the midpoint of left and right ecm_u6 points
		Dental Arch Height ex6, Upper|^d^	Distance from the midpoint of a(ANS)-alv (PNS) to the plane formed by ale, bilateral ecm_u6 points
		Mandibular Body Length L|^d^	Distance from left ml to left lg
		Mandibular Body Length R|^d^	Distance from right ml to right lg
		AgL-AgR|^d^	Distance between left and right ag points
		GoL-GoR|^d^	Distance between left and right go points
		JuL-JuR|^d^	Distance between left and right ju points
		ZmL-ZmR|^d^	Distance between left and right zm points
		ZyL-ZyR|^d^	Distance between left and right zy points
		Piriform Apertura Width|^d^	Distance between left and right al points
		Overbite|^d^	The difference in distance from u1 and l1 to HP ^2^
		Overjet|^d^	The difference in distance from u1 and l1 to CP ^2^
		N-Me|^d^	Distance from n to me; projected on SP
		Gn-A|^d^	Distance from gn to ss(A); projected on SP
		N-ANS|^d^	Distance from n to a(ANS); projected on SP
Soft Tissue	Angular	t’-n’-mp’|°	t’(mid)-n’-mp’ angle; projected on SP ^4^
		t’-n’-sm’|°	t’(mid)-n’-sm’ angle; projected on SP ^4^
		Mouth Canting|°	Angle between HP and the line connecting bilateral ch’ points; projected on CP
		Nasolabial Angle |°	c’-sn’-ls’ angle; projected on SP
		Upper Lip Angle |°	Angle between ls’-sn’ and CP; projected on SP
		g’-sn’-pg’|°	g’-sn’-pg’ angle; projected on SP ^5^
		g’-se’-pn’|°	g’-se’-pg’ angle; projected on SP
		Eyelid Canting|°	Angle between HP and the line connecting bilateral ps’ points; projected on CP
		Eye Canting|°	Angle between the midpoints of bilateral en’ and ex’ points; projected on CP
	Linear	n’-mp’|^d^	Distance from n’ to mp’; projected on SP
		n’-sm’|^d^	Distance from n’ to sm’; projected on SP
		mp’-sm’|^d^	Distance from mp’ to sm’; projected on SP
		pn’-sn’|^d^	Distance from pn’ to sn’; projected on SP
		Upper Lip Length|^d^	Distance from sn’ to sto’; projected on SP
		Upper Vermilion Width|^d^	Distance from ls’ to sto’; projected on SP
		pg’-SP|^d^	Distance from pg’ to SP
		U1 exposure|^d^	The difference in distance from u1 and sto’ points to HP ^1^
		Lower 1/3 Height|^d^	The difference in distance from sn’ and me’ to HP
		Facial Height_n’|^d^	The difference in distance from n’ and me’ to HP
		Inner Canthic Diameter|^d^	Distance between left and right en’ points
		g’-TVL|^d^	Distance from g’ to TVL
		pn’-TVL|^d^	Distance from pn’ to TVL
		mp’-TVL|^d^	Distance from mp’ to TVL
		li’-TVL|^d^	Distance from li’ to TVL
		sm’-TVL|^d^	Distance from sm’ to TVL
		pg’-TVL|^d^	Distance from pg’ to TVL
		gn’-TVL|^d^	Distance from gn’ to TVL
Airway	Linear	H-MP|^d^	Distance from h to MP
		H-C|^d^	Distance from h to the midpoint of c2 and c3
	Areal	Airway, Mean|^a 6^	The average cross-sectional area of the airway enclosed by the planes parallel to FH and passing through alv(PNS) and c3
		Velopharynx, Mean|^a^	The average cross-sectional area of the airway enclosed by the planes parallel to FH and passing through alv(PNS) and u
		Glossopharynx, Mean|^a^	The average cross-sectional area of the airway enclosed by the planes parallel to FH and passing through u and c3
		Airway, Min|^a^	The minimum cross-sectional area (1 mm per step) of the airway enclosed by the planes parallel to FH and passing through alv(PNS) and c3
	Voluminal	Airway|^v 7^	The volume of the airway enclosed by the planes parallel to FH and passing through alv(PNS) and c3
		Velopharynx|^v^	The volume of the airway enclosed by the planes parallel to FH and passing through alv(PNS) and u
		Glossopharynx|^v^	The volume of the airway enclosed by the planes parallel to FH and passing through u and c3

^1^|° represents that the parameter is angular. ^2^ Upper incisor axis refers to the line u1-apu1; u1 is the midpoint of u1d_L, u1d_R, u1m_L, and u1m_R. The lower incisor axis is the same. ^3^|^d^ represents that the parameter is linear. ^4^ t’(mid) is the midpoint of t’_L, t’_R. ^5^ We use the supplementary angle of g’-sn’-pg’ to make sure it is acute. ^6^|^a^ represents that the parameter is areal. ^7^|^v^ represents that the parameter is voluminal.

**Table 4:** The inter-examiner and intra-examiner ICCs for each landmark. ICCs <0.75 are marked in green. ICCs with lower bound of 95% confidence interval <0.50 are labeled in red.

Region	Landmarks	Inter-Examiner						Examiner 1						Examiner 2					
		L ^1^ ICC	95% CI	P ^2^ ICC	95% CI	S ^3^ ICC	95% CI	L ICC	95% CI	P ICC	95% CI	S ICC	95% CI	L ICC	95% CI	P ICC	95% CI	S ICC	95% CI
Cranial	s	0.99	[0.98,1.00]	1.00	[0.99,1.00]	1.00	[1.00,1.00]	1.00	[0.99,1.00]	1.00	[0.99,1.00]	1.00	[0.99,1.00]	0.98	[0.94,0.99]	1.00	[0.99,1.00]	1.00	[1.00,1.00]
	ba	1.00	[1.00,1.00]	1.00	[0.98,1.00]	1.00	[0.99,1.00]	1.00	[1.00,1.00]	1.00	[1.00,1.00]	1.00	[1.00,1.00]	1.00	[0.99,1.00]	1.00	[1.00,1.00]	1.00	[1.00,1.00]
	n	1.00	[1.00,1.00]	1.00	[0.99,1.00]	1.00	[0.93,1.00]	1.00	[1.00,1.00]	1.00	[1.00,1.00]	1.00	[0.99,1.00]	1.00	[1.00,1.00]	1.00	[1.00,1.00]	1.00	[0.99,1.00]
	a(ANS)	1.00	[1.00,1.00]	1.00	[1.00,1.00]	1.00	[0.98,1.00]	1.00	[1.00,1.00]	1.00	[1.00,1.00]	1.00	[1.00,1.00]	1.00	[1.00,1.00]	1.00	[1.00,1.00]	1.00	[1.00,1.00]
	ss(A)	1.00	[1.00,1.00]	1.00	[1.00,1.00]	0.99	[0.97,1.00]	1.00	[1.00,1.00]	1.00	[1.00,1.00]	0.99	[0.98,1.00]	1.00	[1.00,1.00]	1.00	[1.00,1.00]	0.99	[0.97,1.00]
	alv(PNS) ^4^	1.00	[1.00,1.00]	1.00	[1.00,1.00]	1.00	[0.99,1.00]	1.00	[1.00,1.00]	1.00	[1.00,1.00]	1.00	[1.00,1.00]	1.00	[0.99,1.00]	1.00	[1.00,1.00]	1.00	[0.99,1.00]
	ale	1.00	[1.00,1.00]	1.00	[0.96,1.00]	0.99	[0.78,1.00]	1.00	[1.00,1.00]	1.00	[1.00,1.00]	1.00	[0.99,1.00]	1.00	[1.00,1.00]	1.00	[1.00,1.00]	1.00	[0.99,1.00]
	ol	1.00	[1.00,1.00]	1.00	[1.00,1.00]	1.00	[0.99,1.00]	1.00	[1.00,1.00]	1.00	[1.00,1.00]	1.00	[0.99,1.00]	1.00	[1.00,1.00]	1.00	[1.00,1.00]	0.99	[0.98,1.00]
	po_L	0.96	[0.73,0.99]	0.99	[0.98,1.00]	1.00	[1.00,1.00]	0.99	[0.98,1.00]	1.00	[0.99,1.00]	1.00	[1.00,1.00]	0.98	[0.95,0.99]	1.00	[0.99,1.00]	1.00	[0.99,1.00]
	po_R	0.97	[0.90,0.99]	0.99	[0.95,1.00]	1.00	[1.00,1.00]	0.99	[0.97,1.00]	1.00	[0.99,1.00]	1.00	[1.00,1.00]	0.97	[0.91,0.99]	1.00	[0.99,1.00]	1.00	[1.00,1.00]
	ms_L	1.00	[0.99,1.00]	1.00	[0.99,1.00]	1.00	[1.00,1.00]	1.00	[0.99,1.00]	1.00	[0.99,1.00]	1.00	[1.00,1.00]	1.00	[0.99,1.00]	1.00	[0.99,1.00]	1.00	[1.00,1.00]
	ms_R	1.00	[0.99,1.00]	1.00	[0.99,1.00]	1.00	[1.00,1.00]	1.00	[1.00,1.00]	1.00	[0.99,1.00]	1.00	[1.00,1.00]	1.00	[0.99,1.00]	1.00	[0.99,1.00]	1.00	[1.00,1.00]
	or_L	0.96	[0.15,0.99]	1.00	[0.99,1.00]	1.00	[1.00,1.00]	0.99	[0.96,0.99]	1.00	[0.99,1.00]	1.00	[1.00,1.00]	0.99	[0.98,1.00]	1.00	[0.99,1.00]	1.00	[0.99,1.00]
	or_R	0.99	[0.98,1.00]	1.00	[0.84,1.00]	1.00	[0.80,1.00]	0.98	[0.95,0.99]	1.00	[0.99,1.00]	1.00	[0.99,1.00]	0.98	[0.95,0.99]	1.00	[0.99,1.00]	1.00	[0.99,1.00]
	al_L	1.00	[0.99,1.00]	1.00	[0.99,1.00]	0.99	[0.93,1.00]	1.00	[1.00,1.00]	1.00	[0.99,1.00]	0.98	[0.94,0.99]	1.00	[1.00,1.00]	1.00	[1.00,1.00]	0.99	[0.97,1.00]
	al_R	1.00	[1.00,1.00]	1.00	[0.97,1.00]	0.99	[0.69,1.00]	1.00	[1.00,1.00]	1.00	[0.99,1.00]	0.98	[0.95,0.99]	1.00	[1.00,1.00]	1.00	[1.00,1.00]	0.99	[0.98,1.00]
	zm_L	0.99	[0.97,1.00]	0.99	[0.96,1.00]	1.00	[0.99,1.00]	0.98	[0.95,0.99]	1.00	[0.99,1.00]	0.99	[0.97,1.00]	0.98	[0.94,0.99]	0.99	[0.98,1.00]	0.99	[0.96,0.99]
	zm_R	1.00	[0.99,1.00]	1.00	[0.98,1.00]	1.00	[0.99,1.00]	0.99	[0.96,1.00]	0.99	[0.97,1.00]	1.00	[0.99,1.00]	0.98	[0.94,0.99]	1.00	[0.99,1.00]	1.00	[0.99,1.00]
	ju_L	1.00	[1.00,1.00]	0.99	[0.85,1.00]	1.00	[0.98,1.00]	1.00	[1.00,1.00]	0.99	[0.98,1.00]	1.00	[0.99,1.00]	1.00	[0.99,1.00]	1.00	[0.99,1.00]	1.00	[1.00,1.00]
	ju_R	1.00	[1.00,1.00]	1.00	[0.99,1.00]	1.00	[1.00,1.00]	1.00	[0.99,1.00]	0.99	[0.98,1.00]	1.00	[1.00,1.00]	1.00	[1.00,1.00]	1.00	[0.99,1.00]	1.00	[1.00,1.00]
	zy_L	0.99	[0.94,1.00]	0.87	[0.30,0.96]	0.99	[0.97,1.00]	0.99	[0.97,1.00]	0.90	[0.72,0.96]	0.99	[0.96,1.00]	1.00	[0.99,1.00]	0.95	[0.84,0.98]	0.98	[0.95,0.99]
	zy_R	0.99	[0.98,1.00]	0.95	[0.85,0.98]	0.99	[0.96,1.00]	0.99	[0.96,1.00]	0.90	[0.72,0.96]	0.99	[0.96,1.00]	0.97	[0.92,0.99]	0.83	[0.56,0.94]	0.96	[0.89,0.99]
	ecm_u6_L	0.99	[0.49,1.00]	1.00	[0.98,1.00]	1.00	[0.84,1.00]	1.00	[0.99,1.00]	1.00	[1.00,1.00]	1.00	[0.99,1.00]	0.99	[0.98,1.00]	1.00	[0.99,1.00]	1.00	[0.99,1.00]
	ecm_u6_R	0.99	[0.44,1.00]	1.00	[0.99,1.00]	0.99	[0.50,1.00]	1.00	[0.99,1.00]	1.00	[0.99,1.00]	1.00	[1.00,1.00]	1.00	[0.99,1.00]	1.00	[1.00,1.00]	1.00	[0.99,1.00]
	ecm_u7_L	0.99	[0.68,1.00]	0.99	[0.93,1.00]	0.96	[0.12,0.99]	1.00	[1.00,1.00]	1.00	[0.99,1.00]	0.99	[0.97,1.00]	1.00	[1.00,1.00]	1.00	[0.99,1.00]	1.00	[0.99,1.00]
	ecm_u7_R	0.99	[0.38,1.00]	0.99	[0.92,1.00]	0.96	[0.04,0.99]	1.00	[1.00,1.00]	0.99	[0.97,1.00]	1.00	[0.99,1.00]	1.00	[1.00,1.00]	1.00	[1.00,1.00]	1.00	[0.99,1.00]
	enm_u7_L	0.98	[0.33,1.00]	0.99	[0.97,1.00]	0.94	[0.25,0.99]	1.00	[0.99,1.00]	1.00	[0.99,1.00]	0.99	[0.97,1.00]	1.00	[0.99,1.00]	1.00	[0.98,1.00]	0.99	[0.98,1.00]
	enm_u7_R	0.98	[0.36,1.00]	1.00	[0.99,1.00]	0.94	[0.12,0.99]	1.00	[1.00,1.00]	0.99	[0.97,1.00]	0.99	[0.96,1.00]	1.00	[1.00,1.00]	1.00	[0.99,1.00]	1.00	[0.99,1.00]
Mandibular	id	1.00	[1.00,1.00]	1.00	[1.00,1.00]	0.98	[0.69,0.99]	1.00	[1.00,1.00]	1.00	[1.00,1.00]	0.99	[0.98,1.00]	1.00	[1.00,1.00]	1.00	[1.00,1.00]	0.99	[0.98,1.00]
	li	1.00	[1.00,1.00]	1.00	[0.99,1.00]	1.00	[0.99,1.00]	1.00	[1.00,1.00]	1.00	[0.99,1.00]	1.00	[1.00,1.00]	1.00	[1.00,1.00]	1.00	[0.99,1.00]	0.99	[0.98,1.00]
	sm(B)	1.00	[0.99,1.00]	1.00	[0.99,1.00]	0.89	[0.59,0.97]	1.00	[1.00,1.00]	1.00	[1.00,1.00]	0.97	[0.91,0.99]	1.00	[0.99,1.00]	1.00	[0.98,1.00]	0.86	[0.62,0.95]
	pg	1.00	[0.98,1.00]	1.00	[0.98,1.00]	0.98	[0.91,0.99]	1.00	[0.99,1.00]	1.00	[1.00,1.00]	0.98	[0.95,0.99]	0.99	[0.98,1.00]	1.00	[1.00,1.00]	0.99	[0.98,1.00]
	gn	1.00	[0.98,1.00]	0.99	[0.28,1.00]	0.99	[0.57,1.00]	1.00	[0.99,1.00]	1.00	[1.00,1.00]	1.00	[0.99,1.00]	0.99	[0.98,1.00]	1.00	[1.00,1.00]	1.00	[0.99,1.00]
	me	0.99	[0.97,1.00]	0.98	[0.20,1.00]	0.99	[0.62,1.00]	1.00	[0.99,1.00]	1.00	[0.99,1.00]	1.00	[1.00,1.00]	0.99	[0.98,1.00]	1.00	[0.99,1.00]	1.00	[0.99,1.00]
	ge	1.00	[0.99,1.00]	1.00	[0.99,1.00]	0.99	[0.98,1.00]	1.00	[1.00,1.00]	1.00	[0.99,1.00]	0.99	[0.97,1.00]	1.00	[0.99,1.00]	1.00	[0.99,1.00]	0.98	[0.95,0.99]
	col_L	0.97	[0.92,0.99]	0.99	[0.88,1.00]	1.00	[0.98,1.00]	0.98	[0.94,0.99]	1.00	[0.99,1.00]	1.00	[1.00,1.00]	0.99	[0.97,1.00]	1.00	[0.99,1.00]	1.00	[1.00,1.00]
	col_R	0.99	[0.96,1.00]	0.99	[0.76,1.00]	1.00	[0.96,1.00]	0.99	[0.98,1.00]	1.00	[1.00,1.00]	1.00	[1.00,1.00]	0.99	[0.96,1.00]	0.99	[0.97,1.00]	1.00	[1.00,1.00]
	cdl_L	1.00	[0.99,1.00]	1.00	[0.99,1.00]	0.99	[0.98,1.00]	1.00	[1.00,1.00]	1.00	[0.99,1.00]	1.00	[0.99,1.00]	1.00	[1.00,1.00]	1.00	[1.00,1.00]	1.00	[0.99,1.00]
	cdl_R	1.00	[0.98,1.00]	0.99	[0.96,1.00]	1.00	[0.98,1.00]	1.00	[1.00,1.00]	1.00	[0.99,1.00]	1.00	[0.99,1.00]	1.00	[0.99,1.00]	0.99	[0.98,1.00]	1.00	[0.99,1.00]
	cdm_L	1.00	[1.00,1.00]	1.00	[1.00,1.00]	0.99	[0.98,1.00]	1.00	[1.00,1.00]	1.00	[0.99,1.00]	1.00	[0.99,1.00]	1.00	[1.00,1.00]	1.00	[0.99,1.00]	0.99	[0.98,1.00]
	cdm_R	1.00	[0.99,1.00]	1.00	[0.99,1.00]	0.99	[0.97,1.00]	1.00	[1.00,1.00]	1.00	[1.00,1.00]	1.00	[0.99,1.00]	1.00	[1.00,1.00]	1.00	[0.99,1.00]	1.00	[0.99,1.00]
	go_L	1.00	[0.99,1.00]	0.99	[0.94,1.00]	0.98	[0.52,1.00]	1.00	[0.99,1.00]	0.99	[0.98,1.00]	0.98	[0.93,0.99]	1.00	[0.99,1.00]	1.00	[0.99,1.00]	0.99	[0.95,0.99]
	go_R	1.00	[0.99,1.00]	1.00	[0.97,1.00]	0.98	[0.81,1.00]	1.00	[0.99,1.00]	1.00	[0.99,1.00]	0.98	[0.94,0.99]	1.00	[0.99,1.00]	0.99	[0.98,1.00]	0.96	[0.87,0.98]
	ag_L	1.00	[0.99,1.00]	0.99	[0.98,1.00]	1.00	[0.99,1.00]	0.99	[0.98,1.00]	0.99	[0.98,1.00]	1.00	[0.99,1.00]	1.00	[0.99,1.00]	1.00	[0.99,1.00]	1.00	[0.99,1.00]
	ag_R	0.99	[0.98,1.00]	0.94	[0.48,0.99]	0.96	[0.57,0.99]	1.00	[0.98,1.00]	1.00	[0.99,1.00]	1.00	[0.99,1.00]	1.00	[0.99,1.00]	0.99	[0.98,1.00]	0.99	[0.98,1.00]
	ecm_l6_L	0.98	[0.15,1.00]	0.99	[0.45,1.00]	1.00	[0.99,1.00]	1.00	[1.00,1.00]	1.00	[1.00,1.00]	1.00	[0.99,1.00]	1.00	[0.99,1.00]	1.00	[1.00,1.00]	1.00	[0.99,1.00]
	ecm_l6_R	0.99	[0.28,1.00]	0.99	[0.39,1.00]	1.00	[0.99,1.00]	1.00	[1.00,1.00]	1.00	[0.99,1.00]	0.99	[0.98,1.00]	1.00	[1.00,1.00]	1.00	[0.99,1.00]	0.99	[0.97,1.00]
	ml_L	1.00	[0.96,1.00]	1.00	[0.96,1.00]	1.00	[1.00,1.00]	1.00	[1.00,1.00]	1.00	[1.00,1.00]	1.00	[1.00,1.00]	1.00	[1.00,1.00]	1.00	[1.00,1.00]	1.00	[1.00,1.00]
	ml_R	1.00	[1.00,1.00]	1.00	[0.99,1.00]	1.00	[1.00,1.00]	1.00	[1.00,1.00]	1.00	[1.00,1.00]	1.00	[1.00,1.00]	1.00	[1.00,1.00]	1.00	[1.00,1.00]	1.00	[1.00,1.00]
	lg_L	1.00	[1.00,1.00]	1.00	[1.00,1.00]	1.00	[0.99,1.00]	1.00	[1.00,1.00]	1.00	[1.00,1.00]	1.00	[0.99,1.00]	1.00	[1.00,1.00]	1.00	[0.99,1.00]	0.99	[0.97,1.00]
	lg_R	1.00	[1.00,1.00]	1.00	[1.00,1.00]	1.00	[1.00,1.00]	1.00	[1.00,1.00]	1.00	[1.00,1.00]	1.00	[1.00,1.00]	1.00	[1.00,1.00]	1.00	[1.00,1.00]	1.00	[1.00,1.00]
	enm_l6_L	1.00	[0.95,1.00]	0.98	[0.88,0.99]	0.99	[0.96,1.00]	0.99	[0.98,1.00]	0.94	[0.84,0.98]	0.98	[0.93,0.99]	1.00	[1.00,1.00]	0.99	[0.98,1.00]	0.99	[0.97,1.00]
	enm_l6_R	1.00	[0.81,1.00]	0.96	[0.10,0.99]	1.00	[0.99,1.00]	1.00	[0.99,1.00]	1.00	[0.99,1.00]	1.00	[1.00,1.00]	1.00	[1.00,1.00]	0.98	[0.93,0.99]	0.99	[0.97,1.00]
Teeth	u1d_L	1.00	[0.77,1.00]	1.00	[0.98,1.00]	1.00	[0.79,1.00]	1.00	[1.00,1.00]	1.00	[1.00,1.00]	1.00	[1.00,1.00]	1.00	[1.00,1.00]	1.00	[1.00,1.00]	1.00	[1.00,1.00]
	u1d_R	1.00	[0.35,1.00]	1.00	[1.00,1.00]	0.99	[0.61,1.00]	1.00	[1.00,1.00]	1.00	[1.00,1.00]	1.00	[1.00,1.00]	1.00	[1.00,1.00]	1.00	[1.00,1.00]	1.00	[0.99,1.00]
	u1m_L	1.00	[0.99,1.00]	1.00	[1.00,1.00]	1.00	[0.99,1.00]	1.00	[0.99,1.00]	1.00	[1.00,1.00]	1.00	[1.00,1.00]	1.00	[1.00,1.00]	1.00	[1.00,1.00]	1.00	[1.00,1.00]
	u1m_R	1.00	[0.94,1.00]	1.00	[1.00,1.00]	1.00	[1.00,1.00]	1.00	[0.99,1.00]	1.00	[1.00,1.00]	1.00	[1.00,1.00]	1.00	[1.00,1.00]	1.00	[1.00,1.00]	1.00	[1.00,1.00]
	apu1_L	1.00	[1.00,1.00]	1.00	[1.00,1.00]	1.00	[0.72,1.00]	1.00	[1.00,1.00]	1.00	[1.00,1.00]	1.00	[1.00,1.00]	1.00	[1.00,1.00]	1.00	[1.00,1.00]	1.00	[0.99,1.00]
	apu1_R	1.00	[1.00,1.00]	1.00	[1.00,1.00]	1.00	[0.89,1.00]	1.00	[1.00,1.00]	1.00	[1.00,1.00]	1.00	[1.00,1.00]	1.00	[1.00,1.00]	1.00	[1.00,1.00]	1.00	[1.00,1.00]
	u3_L	1.00	[0.99,1.00]	1.00	[0.99,1.00]	1.00	[1.00,1.00]	0.98	[0.95,0.99]	0.99	[0.97,1.00]	1.00	[0.99,1.00]	1.00	[1.00,1.00]	1.00	[1.00,1.00]	1.00	[1.00,1.00]
	u3_R	1.00	[0.99,1.00]	1.00	[1.00,1.00]	1.00	[1.00,1.00]	1.00	[1.00,1.00]	1.00	[1.00,1.00]	1.00	[1.00,1.00]	1.00	[0.99,1.00]	1.00	[1.00,1.00]	1.00	[1.00,1.00]
	u6_L	1.00	[0.99,1.00]	0.99	[0.98,1.00]	1.00	[0.99,1.00]	1.00	[1.00,1.00]	1.00	[1.00,1.00]	1.00	[1.00,1.00]	1.00	[0.99,1.00]	0.97	[0.92,0.99]	1.00	[0.99,1.00]
	u6_R	1.00	[1.00,1.00]	1.00	[0.99,1.00]	1.00	[1.00,1.00]	1.00	[1.00,1.00]	1.00	[1.00,1.00]	1.00	[1.00,1.00]	1.00	[1.00,1.00]	1.00	[1.00,1.00]	1.00	[1.00,1.00]
	l1d_L	1.00	[1.00,1.00]	1.00	[1.00,1.00]	1.00	[1.00,1.00]	1.00	[1.00,1.00]	1.00	[1.00,1.00]	1.00	[1.00,1.00]	1.00	[1.00,1.00]	1.00	[1.00,1.00]	1.00	[1.00,1.00]
	l1d_R	1.00	[1.00,1.00]	1.00	[1.00,1.00]	1.00	[1.00,1.00]	1.00	[1.00,1.00]	1.00	[1.00,1.00]	1.00	[1.00,1.00]	1.00	[1.00,1.00]	1.00	[1.00,1.00]	1.00	[1.00,1.00]
	l1m_L	1.00	[1.00,1.00]	1.00	[1.00,1.00]	1.00	[1.00,1.00]	1.00	[1.00,1.00]	1.00	[1.00,1.00]	1.00	[1.00,1.00]	1.00	[1.00,1.00]	1.00	[1.00,1.00]	1.00	[1.00,1.00]
	l1m_R	1.00	[1.00,1.00]	1.00	[1.00,1.00]	1.00	[1.00,1.00]	1.00	[1.00,1.00]	1.00	[1.00,1.00]	1.00	[1.00,1.00]	1.00	[1.00,1.00]	1.00	[1.00,1.00]	1.00	[1.00,1.00]
	apl1_L	1.00	[0.99,1.00]	1.00	[1.00,1.00]	1.00	[0.83,1.00]	1.00	[1.00,1.00]	1.00	[1.00,1.00]	1.00	[0.99,1.00]	0.99	[0.97,1.00]	1.00	[0.99,1.00]	1.00	[0.99,1.00]
	apl1_R	1.00	[0.99,1.00]	1.00	[1.00,1.00]	0.99	[0.71,1.00]	1.00	[1.00,1.00]	1.00	[1.00,1.00]	1.00	[0.99,1.00]	0.99	[0.96,1.00]	1.00	[0.99,1.00]	1.00	[0.98,1.00]
	l3_L	1.00	[1.00,1.00]	1.00	[1.00,1.00]	1.00	[1.00,1.00]	1.00	[1.00,1.00]	1.00	[1.00,1.00]	1.00	[1.00,1.00]	1.00	[1.00,1.00]	1.00	[1.00,1.00]	1.00	[1.00,1.00]
	l3_R	1.00	[1.00,1.00]	1.00	[1.00,1.00]	1.00	[1.00,1.00]	1.00	[0.99,1.00]	1.00	[1.00,1.00]	1.00	[1.00,1.00]	1.00	[1.00,1.00]	1.00	[1.00,1.00]	1.00	[1.00,1.00]
	l6_L	1.00	[1.00,1.00]	1.00	[1.00,1.00]	1.00	[1.00,1.00]	1.00	[1.00,1.00]	1.00	[1.00,1.00]	1.00	[0.99,1.00]	1.00	[1.00,1.00]	1.00	[1.00,1.00]	1.00	[1.00,1.00]
	l6_R	1.00	[0.99,1.00]	1.00	[1.00,1.00]	1.00	[0.97,1.00]	0.99	[0.97,1.00]	1.00	[0.99,1.00]	1.00	[0.99,1.00]	1.00	[0.99,1.00]	1.00	[1.00,1.00]	1.00	[0.99,1.00]
Soft Tissue	g’	1.00	[0.99,1.00]	1.00	[0.99,1.00]	0.91	[0.02,0.98]	0.99	[0.97,1.00]	1.00	[0.99,1.00]	0.97	[0.92,0.99]	1.00	[0.99,1.00]	1.00	[1.00,1.00]	0.98	[0.94,0.99]
	n’	1.00	[0.99,1.00]	1.00	[0.99,1.00]	1.00	[0.99,1.00]	1.00	[1.00,1.00]	1.00	[0.99,1.00]	0.99	[0.96,1.00]	1.00	[0.99,1.00]	1.00	[0.99,1.00]	1.00	[0.98,1.00]
	se’	1.00	[0.99,1.00]	1.00	[0.96,1.00]	0.98	[0.45,0.99]	1.00	[1.00,1.00]	1.00	[1.00,1.00]	0.99	[0.98,1.00]	1.00	[1.00,1.00]	1.00	[1.00,1.00]	0.99	[0.98,1.00]
	pn’	1.00	[1.00,1.00]	1.00	[1.00,1.00]	0.98	[0.48,1.00]	1.00	[1.00,1.00]	1.00	[1.00,1.00]	0.99	[0.97,1.00]	1.00	[1.00,1.00]	1.00	[0.99,1.00]	0.94	[0.83,0.98]
	sn’	1.00	[1.00,1.00]	1.00	[0.69,1.00]	0.98	[0.28,1.00]	1.00	[1.00,1.00]	1.00	[1.00,1.00]	1.00	[0.99,1.00]	1.00	[1.00,1.00]	1.00	[0.99,1.00]	1.00	[0.99,1.00]
	c’	1.00	[1.00,1.00]	1.00	[1.00,1.00]	1.00	[1.00,1.00]	1.00	[1.00,1.00]	1.00	[0.99,1.00]	1.00	[1.00,1.00]	1.00	[1.00,1.00]	1.00	[1.00,1.00]	1.00	[1.00,1.00]
	ls’	1.00	[1.00,1.00]	1.00	[1.00,1.00]	1.00	[0.99,1.00]	1.00	[1.00,1.00]	1.00	[1.00,1.00]	0.99	[0.98,1.00]	1.00	[1.00,1.00]	1.00	[1.00,1.00]	0.99	[0.98,1.00]
	mp’	1.00	[1.00,1.00]	1.00	[1.00,1.00]	1.00	[0.99,1.00]	1.00	[0.99,1.00]	1.00	[1.00,1.00]	0.99	[0.98,1.00]	1.00	[1.00,1.00]	1.00	[1.00,1.00]	1.00	[0.99,1.00]
	sto’	1.00	[1.00,1.00]	0.99	[0.97,1.00]	1.00	[0.99,1.00]	1.00	[0.99,1.00]	0.99	[0.97,1.00]	1.00	[0.99,1.00]	1.00	[1.00,1.00]	1.00	[0.98,1.00]	1.00	[1.00,1.00]
	li’	1.00	[0.97,1.00]	1.00	[1.00,1.00]	1.00	[0.99,1.00]	1.00	[0.99,1.00]	1.00	[0.99,1.00]	0.99	[0.98,1.00]	0.99	[0.98,1.00]	1.00	[1.00,1.00]	1.00	[0.98,1.00]
	sm’	1.00	[0.99,1.00]	1.00	[0.99,1.00]	0.93	[0.81,0.98]	1.00	[0.99,1.00]	1.00	[0.99,1.00]	0.96	[0.87,0.98]	0.99	[0.98,1.00]	0.99	[0.97,1.00]	0.85	[0.60,0.95]
	me’	1.00	[0.99,1.00]	0.99	[0.90,1.00]	0.99	[0.95,1.00]	0.99	[0.98,1.00]	0.97	[0.92,0.99]	1.00	[0.99,1.00]	1.00	[0.99,1.00]	1.00	[0.98,1.00]	1.00	[0.99,1.00]
	pg’	1.00	[0.98,1.00]	0.83	[0.25,0.95]	0.43	[−0.11,0.79]	1.00	[0.99,1.00]	1.00	[0.99,1.00]	0.96	[0.89,0.99]	1.00	[0.99,1.00]	0.99	[0.98,1.00]	0.97	[0.91,0.99]
	gn’	1.00	[0.99,1.00]	0.91	[0.27,0.98]	0.83	[−0.01,0.96]	1.00	[0.99,1.00]	0.99	[0.97,1.00]	0.98	[0.94,0.99]	1.00	[0.99,1.00]	0.99	[0.98,1.00]	0.99	[0.98,1.00]
	al’_L	1.00	[0.99,1.00]	0.99	[0.48,1.00]	0.99	[0.98,1.00]	1.00	[1.00,1.00]	1.00	[0.99,1.00]	1.00	[0.99,1.00]	1.00	[0.99,1.00]	1.00	[0.99,1.00]	0.98	[0.94,0.99]
	al’_R	1.00	[0.96,1.00]	0.99	[0.65,1.00]	1.00	[0.99,1.00]	1.00	[1.00,1.00]	1.00	[0.99,1.00]	0.99	[0.98,1.00]	1.00	[1.00,1.00]	1.00	[0.99,1.00]	0.99	[0.96,1.00]
	ma’_L	1.00	[0.99,1.00]	1.00	[0.99,1.00]	0.99	[0.98,1.00]	1.00	[0.98,1.00]	1.00	[0.99,1.00]	0.99	[0.97,1.00]	1.00	[0.98,1.00]	0.99	[0.98,1.00]	0.99	[0.96,0.99]
	ma’_R	1.00	[0.96,1.00]	1.00	[0.98,1.00]	0.99	[0.80,1.00]	0.99	[0.97,1.00]	0.99	[0.98,1.00]	0.99	[0.98,1.00]	0.99	[0.98,1.00]	0.99	[0.98,1.00]	0.98	[0.95,0.99]
	ac’_L	1.00	[0.99,1.00]	1.00	[0.99,1.00]	0.99	[0.97,1.00]	1.00	[0.99,1.00]	1.00	[0.99,1.00]	0.98	[0.95,0.99]	1.00	[1.00,1.00]	1.00	[0.99,1.00]	0.98	[0.95,0.99]
	ac’_R	1.00	[1.00,1.00]	1.00	[0.99,1.00]	1.00	[0.99,1.00]	1.00	[0.99,1.00]	1.00	[0.99,1.00]	0.99	[0.96,1.00]	1.00	[0.99,1.00]	1.00	[0.99,1.00]	0.99	[0.95,0.99]
	sbal’_L	0.99	[0.75,1.00]	1.00	[0.97,1.00]	0.99	[0.83,1.00]	1.00	[0.99,1.00]	1.00	[0.99,1.00]	1.00	[0.99,1.00]	1.00	[0.99,1.00]	1.00	[1.00,1.00]	1.00	[0.99,1.00]
	sbal’_R	0.95	[0.05,0.99]	0.99	[0.32,1.00]	0.98	[0.26,1.00]	1.00	[0.99,1.00]	1.00	[1.00,1.00]	1.00	[1.00,1.00]	0.99	[0.97,1.00]	1.00	[0.98,1.00]	1.00	[0.99,1.00]
	mc’_L	0.99	[0.43,1.00]	1.00	[0.88,1.00]	0.99	[0.83,1.00]	1.00	[0.99,1.00]	1.00	[1.00,1.00]	1.00	[0.99,1.00]	1.00	[0.99,1.00]	1.00	[0.99,1.00]	0.99	[0.97,1.00]
	mc’_R	0.99	[0.62,1.00]	1.00	[0.84,1.00]	0.99	[0.95,1.00]	1.00	[0.99,1.00]	1.00	[1.00,1.00]	0.99	[0.98,1.00]	1.00	[1.00,1.00]	1.00	[1.00,1.00]	0.99	[0.97,1.00]
	cs’_L	0.99	[0.54,1.00]	1.00	[1.00,1.00]	1.00	[0.93,1.00]	1.00	[0.99,1.00]	1.00	[1.00,1.00]	1.00	[0.99,1.00]	1.00	[1.00,1.00]	1.00	[1.00,1.00]	1.00	[1.00,1.00]
	cs’_R	1.00	[0.77,1.00]	1.00	[1.00,1.00]	0.99	[0.86,1.00]	1.00	[0.99,1.00]	1.00	[1.00,1.00]	1.00	[0.99,1.00]	1.00	[0.99,1.00]	1.00	[1.00,1.00]	0.99	[0.98,1.00]
	vs’_L	0.99	[0.98,1.00]	1.00	[1.00,1.00]	0.98	[0.17,1.00]	1.00	[0.99,1.00]	1.00	[1.00,1.00]	1.00	[0.99,1.00]	1.00	[0.99,1.00]	1.00	[1.00,1.00]	0.99	[0.98,1.00]
	vs’_R	1.00	[0.99,1.00]	1.00	[1.00,1.00]	0.97	[0.12,0.99]	1.00	[0.99,1.00]	1.00	[1.00,1.00]	1.00	[0.99,1.00]	0.99	[0.97,1.00]	1.00	[1.00,1.00]	1.00	[0.99,1.00]
	ch’_L	0.97	[0.92,0.99]	1.00	[0.89,1.00]	0.99	[0.93,1.00]	0.98	[0.95,0.99]	1.00	[0.99,1.00]	1.00	[0.98,1.00]	0.98	[0.93,0.99]	0.99	[0.97,1.00]	0.99	[0.97,1.00]
	ch’_R	0.99	[0.98,1.00]	1.00	[1.00,1.00]	1.00	[0.98,1.00]	1.00	[0.99,1.00]	1.00	[0.99,1.00]	1.00	[0.99,1.00]	1.00	[0.99,1.00]	1.00	[1.00,1.00]	1.00	[0.99,1.00]
	vi’_L	0.97	[0.07,1.00]	1.00	[0.99,1.00]	1.00	[0.98,1.00]	0.99	[0.98,1.00]	1.00	[0.99,1.00]	0.99	[0.98,1.00]	0.99	[0.97,1.00]	1.00	[0.99,1.00]	0.99	[0.98,1.00]
	vi’_R	0.99	[0.98,1.00]	1.00	[1.00,1.00]	1.00	[0.99,1.00]	0.99	[0.97,1.00]	1.00	[0.99,1.00]	1.00	[0.99,1.00]	0.99	[0.96,1.00]	1.00	[0.99,1.00]	1.00	[0.99,1.00]
	obs’_L	1.00	[1.00,1.00]	1.00	[0.99,1.00]	1.00	[0.99,1.00]	1.00	[1.00,1.00]	1.00	[0.99,1.00]	1.00	[0.99,1.00]	1.00	[0.99,1.00]	1.00	[0.98,1.00]	0.99	[0.98,1.00]
	obs’_R	1.00	[0.99,1.00]	1.00	[1.00,1.00]	1.00	[0.99,1.00]	1.00	[0.99,1.00]	1.00	[1.00,1.00]	1.00	[0.99,1.00]	1.00	[0.99,1.00]	1.00	[0.99,1.00]	0.99	[0.98,1.00]
	t’_L	0.99	[0.64,1.00]	1.00	[0.98,1.00]	1.00	[0.99,1.00]	1.00	[0.99,1.00]	1.00	[0.99,1.00]	1.00	[0.99,1.00]	0.99	[0.98,1.00]	1.00	[1.00,1.00]	1.00	[0.99,1.00]
	t’_R	0.99	[0.96,1.00]	1.00	[1.00,1.00]	1.00	[0.99,1.00]	0.99	[0.98,1.00]	1.00	[0.99,1.00]	1.00	[1.00,1.00]	0.97	[0.92,0.99]	1.00	[0.99,1.00]	1.00	[1.00,1.00]
	it’_L	0.99	[0.90,1.00]	0.99	[0.71,1.00]	1.00	[1.00,1.00]	1.00	[0.99,1.00]	1.00	[1.00,1.00]	1.00	[1.00,1.00]	0.98	[0.94,0.99]	0.99	[0.98,1.00]	1.00	[0.99,1.00]
	it’_R	0.99	[0.96,1.00]	1.00	[0.99,1.00]	1.00	[0.99,1.00]	1.00	[0.99,1.00]	1.00	[0.99,1.00]	1.00	[1.00,1.00]	0.98	[0.93,0.99]	0.99	[0.98,1.00]	1.00	[1.00,1.00]
	obi’_L	1.00	[0.91,1.00]	1.00	[0.97,1.00]	1.00	[0.91,1.00]	1.00	[0.99,1.00]	1.00	[0.99,1.00]	1.00	[0.99,1.00]	1.00	[1.00,1.00]	0.99	[0.98,1.00]	1.00	[0.99,1.00]
	obi’_R	1.00	[0.99,1.00]	0.99	[0.98,1.00]	1.00	[0.98,1.00]	0.99	[0.98,1.00]	0.99	[0.98,1.00]	1.00	[0.99,1.00]	1.00	[0.99,1.00]	0.99	[0.98,1.00]	1.00	[0.99,1.00]
	ps’_L	0.99	[0.97,1.00]	1.00	[0.99,1.00]	1.00	[1.00,1.00]	0.98	[0.95,0.99]	1.00	[1.00,1.00]	1.00	[1.00,1.00]	0.98	[0.95,0.99]	1.00	[0.99,1.00]	1.00	[1.00,1.00]
	ps’_R	0.97	[0.91,0.99]	1.00	[0.98,1.00]	1.00	[1.00,1.00]	0.98	[0.94,0.99]	1.00	[0.99,1.00]	1.00	[1.00,1.00]	0.99	[0.98,1.00]	1.00	[0.99,1.00]	1.00	[1.00,1.00]
	en’_L	1.00	[0.98,1.00]	1.00	[0.99,1.00]	1.00	[0.81,1.00]	0.99	[0.96,1.00]	1.00	[1.00,1.00]	1.00	[0.99,1.00]	0.97	[0.93,0.99]	0.99	[0.98,1.00]	1.00	[0.99,1.00]
	en’_R	0.99	[0.97,1.00]	1.00	[0.99,1.00]	1.00	[0.90,1.00]	1.00	[1.00,1.00]	1.00	[1.00,1.00]	1.00	[0.99,1.00]	0.98	[0.93,0.99]	0.99	[0.98,1.00]	1.00	[1.00,1.00]
	ex’_L	0.99	[0.96,1.00]	0.99	[0.97,1.00]	1.00	[0.99,1.00]	1.00	[0.99,1.00]	1.00	[0.99,1.00]	1.00	[0.99,1.00]	0.96	[0.89,0.99]	0.98	[0.94,0.99]	0.99	[0.97,1.00]
	ex’_R	0.99	[0.98,1.00]	1.00	[0.98,1.00]	1.00	[1.00,1.00]	0.99	[0.98,1.00]	1.00	[0.99,1.00]	1.00	[0.99,1.00]	0.99	[0.95,0.99]	0.99	[0.97,1.00]	1.00	[0.99,1.00]
	zy’_L	0.97	[0.88,0.99]	0.66	[0.02,0.89]	0.80	[0.10,0.95]	0.97	[0.92,0.99]	0.81	[0.53,0.93]	0.89	[0.71,0.96]	0.97	[0.91,0.99]	0.67	[0.26,0.88]	0.86	[0.63,0.95]
	zy’_R	0.99	[0.96,0.99]	0.84	[0.56,0.95]	0.79	[−0.05,0.95]	0.97	[0.92,0.99]	0.74	[0.38,0.90]	0.90	[0.73,0.97]	0.95	[0.87,0.98]	0.74	[0.38,0.90]	0.91	[0.74,0.97]
Airway	alv(PNS) ^4^	1.00	[1.00,1.00]	1.00	[1.00,1.00]	1.00	[0.99,1.00]	1.00	[1.00,1.00]	1.00	[1.00,1.00]	1.00	[1.00,1.00]	1.00	[0.99,1.00]	1.00	[1.00,1.00]	1.00	[0.99,1.00]
	u	1.00	[0.99,1.00]	1.00	[0.99,1.00]	0.97	[0.90,0.99]	1.00	[0.99,1.00]	1.00	[0.99,1.00]	0.99	[0.97,1.00]	1.00	[0.99,1.00]	0.99	[0.96,1.00]	0.91	[0.76,0.97]
	h	1.00	[0.99,1.00]	1.00	[1.00,1.00]	1.00	[0.99,1.00]	1.00	[1.00,1.00]	1.00	[1.00,1.00]	1.00	[0.99,1.00]	1.00	[1.00,1.00]	1.00	[0.99,1.00]	1.00	[1.00,1.00]
	c3	1.00	[1.00,1.00]	1.00	[0.98,1.00]	1.00	[1.00,1.00]	1.00	[0.99,1.00]	1.00	[1.00,1.00]	1.00	[1.00,1.00]	1.00	[1.00,1.00]	1.00	[1.00,1.00]	1.00	[1.00,1.00]
	c2	1.00	[0.99,1.00]	1.00	[1.00,1.00]	1.00	[1.00,1.00]	1.00	[1.00,1.00]	1.00	[0.99,1.00]	1.00	[1.00,1.00]	1.00	[1.00,1.00]	1.00	[1.00,1.00]	1.00	[1.00,1.00]
	epi	0.99	[0.96,0.99]	1.00	[0.99,1.00]	0.96	[0.84,0.99]	1.00	[1.00,1.00]	1.00	[0.99,1.00]	0.99	[0.97,1.00]	0.99	[0.98,1.00]	0.99	[0.98,1.00]	0.98	[0.93,0.99]

^1^ Values of left dimension in LPS coordinate system. ^2^ Values of posterior dimension in LPS coordinate system. ^3^ Values of superior dimension in LPS coordinate system. ^4^ Point alv(PNS) was included in both cranial and airway sets.

**Table 5:** The inter-examiner and intra-examiner ICCs for each parameter. ICCs <0.75 are marked in green. ICCs with lower bound of 95% confidence interval <0.50 are labeled in red.

Classification		Measurement Parameters	Inter-Examiner		Examiner 1		Examiner 2	
			ICC	95% CI	ICC	95% CI	ICC	95% CI
Skeletal	Angular	SNA|° ^1^	0.95	[0.86,0.98]	0.92	[0.77,0.97]	0.93	[0.82,0.98]
		SNB|°	0.99	[0.97,1.00]	0.99	[0.96,1.00]	0.99	[0.97,1.00]
		ANB|°	1.00	[1.00,1.00]	1.00	[1.00,1.00]	1.00	[0.99,1.00]
		NAPg|°	0.99	[0.98,1.00]	1.00	[0.99,1.00]	0.99	[0.98,1.00]
		NSBa|°	0.98	[0.93,0.99]	0.95	[0.87,0.98]	0.98	[0.93,0.99]
		SN-ANSPNS|°	0.97	[0.92,0.99]	0.92	[0.79,0.97]	0.91	[0.75,0.97]
		FMA|°	0.99	[0.98,1.00]	0.99	[0.98,1.00]	1.00	[0.99,1.00]
		Interincisal Angle|°	0.99	[0.97,1.00]	0.98	[0.94,0.99]	0.96	[0.88,0.99]
		U1-SN|°	1.00	[0.99,1.00]	0.99	[0.97,1.00]	0.99	[0.98,1.00]
		L1-APg|°	0.98	[0.94,0.99]	0.98	[0.95,0.99]	0.97	[0.90,0.99]
		Y-axis|°	1.00	[0.98,1.00]	1.00	[0.99,1.00]	0.99	[0.98,1.00]
		OP Tipping|°	1.00	[0.98,1.00]	1.00	[0.98,1.00]	1.00	[0.99,1.00]
		Facial Angle|°	1.00	[1.00,1.00]	1.00	[1.00,1.00]	1.00	[1.00,1.00]
		SN-MP|°	0.99	[0.97,1.00]	0.99	[0.96,1.00]	0.99	[0.97,1.00]
		L1-MP|°	0.92	[0.78,0.97]	0.96	[0.89,0.99]	0.92	[0.77,0.97]
		AB-NPg|°	0.99	[0.97,1.00]	0.99	[0.98,1.00]	0.98	[0.93,0.99]
		Condyle Yaw L|°	0.95	[0.86,0.98]	0.91	[0.76,0.97]	0.96	[0.88,0.99]
		Condyle Yaw R|°	0.93	[0.82,0.98]	0.91	[0.76,0.97]	0.79	[0.48,0.92]
		Maxillary Yawing|°	0.59	[0.13,0.84]	0.83	[0.55,0.94]	0.38	[−0.14,0.74]
		Mastoideus Canting|°	0.97	[0.91,0.99]	0.97	[0.91,0.99]	0.94	[0.83,0.98]
		U6 Canting|°	1.00	[0.99,1.00]	0.99	[0.97,1.00]	0.99	[0.97,1.00]
		U3 Canting|°	0.99	[0.97,1.00]	0.99	[0.97,1.00]	0.98	[0.95,0.99]
		Go Canting|°	0.96	[0.88,0.99]	0.89	[0.69,0.96]	0.71	[0.33,0.89]
	Linear	Pg-SP|^d 2^	0.99	[0.96,0.99]	0.98	[0.93,0.99]	0.94	[0.83,0.98]
		U1-SP|^d^	0.97	[0.92,0.99]	0.95	[0.85,0.98]	0.89	[0.71,0.96]
		L1-SP|^d^	0.98	[0.94,0.99]	0.96	[0.89,0.99]	0.94	[0.82,0.98]
		Dental Arch Width ex6, Upper|^d^	0.93	[0.81,0.98]	0.91	[0.76,0.97]	0.88	[0.69,0.96]
		Dental Arch Length ex6, Upper|^d^	0.99	[0.95,0.99]	0.97	[0.92,0.99]	0.98	[0.93,0.99]
		Dental Arch Height ex6, Upper|^d^	0.99	[0.96,0.99]	0.97	[0.92,0.99]	0.97	[0.90,0.99]
		Mandibular Body Length L|^d^	1.00	[0.99,1.00]	1.00	[1.00,1.00]	0.99	[0.98,1.00]
		Mandibular Body Length R|^d^	1.00	[1.00,1.00]	1.00	[1.00,1.00]	1.00	[0.99,1.00]
		AgL-AgR|^d^	0.94	[0.82,0.98]	0.96	[0.88,0.99]	0.95	[0.85,0.98]
		GoL-GoR|^d^	0.99	[0.96,1.00]	0.98	[0.95,0.99]	0.98	[0.94,0.99]
		JuL-JuR|^d^	1.00	[0.99,1.00]	0.99	[0.98,1.00]	0.99	[0.98,1.00]
		ZmL-ZmR|^d^	0.96	[0.87,0.98]	0.91	[0.75,0.97]	0.84	[0.58,0.94]
		ZyL-ZyR|^d^	0.95	[0.87,0.98]	0.91	[0.76,0.97]	0.92	[0.77,0.97]
		Piriform Apertura Width|^d^	0.96	[0.87,0.98]	0.93	[0.81,0.98]	0.96	[0.90,0.99]
		Overbite|^d^	1.00	[0.99,1.00]	1.00	[0.99,1.00]	1.00	[0.99,1.00]
		Overjet|^d^	1.00	[1.00,1.00]	1.00	[1.00,1.00]	1.00	[1.00,1.00]
		N-Me|^d^	1.00	[0.99,1.00]	1.00	[0.99,1.00]	1.00	[0.99,1.00]
		Gn-A|^d^	0.98	[0.95,0.99]	0.99	[0.97,1.00]	0.98	[0.94,0.99]
		N-ANS|^d^	0.99	[0.98,1.00]	0.99	[0.95,0.99]	0.97	[0.92,0.99]
Soft Tissue	Angular	t’-n’-mp’|°	0.95	[0.87,0.98]	0.93	[0.82,0.98]	0.94	[0.84,0.98]
		t’-n’-sm’|°	0.99	[0.96,1.00]	0.99	[0.96,1.00]	0.98	[0.94,0.99]
		Mouth Canting|°	0.91	[0.75,0.97]	0.93	[0.82,0.98]	0.92	[0.77,0.97]
		Nasolabial Angle |°	0.98	[0.94,0.99]	0.97	[0.92,0.99]	0.95	[0.85,0.98]
		Upper Lip Angle |°	0.97	[0.90,0.99]	0.95	[0.86,0.98]	0.91	[0.75,0.97]
		g’-sn’-pg’|°	0.98	[0.95,0.99]	1.00	[0.99,1.00]	0.99	[0.98,1.00]
		g’-se’-pn’|°	0.89	[0.70,0.96]	0.89	[0.71,0.96]	0.87	[0.65,0.95]
		Eyelid Canting|°	0.85	[0.61,0.95]	0.94	[0.83,0.98]	0.89	[0.70,0.96]
		Eye Canting|°	0.92	[0.78,0.97]	0.88	[0.68,0.96]	0.84	[0.59,0.94]
	Linear	n’-mp’|^d^	0.98	[0.95,0.99]	0.94	[0.83,0.98]	0.97	[0.90,0.99]
		n’-sm’|^d^	0.94	[0.83,0.98]	0.97	[0.91,0.99]	0.85	[0.61,0.95]
		mp’-sm’|^d^	0.83	[0.56,0.94]	0.93	[0.81,0.98]	0.66	[0.23,0.87]
		pn’-sn’|^d^	0.84	[0.59,0.94]	0.85	[0.61,0.95]	0.64	[0.21,0.86]
		Upper Lip Length|^d^	0.95	[0.85,0.98]	0.93	[0.81,0.98]	0.95	[0.87,0.98]
		Upper Vermilion Width|^d^	0.78	[0.45,0.92]	0.70	[0.30,0.89]	0.85	[0.60,0.95]
		pg’-SP|^d^	0.97	[0.90,0.99]	0.95	[0.85,0.98]	0.91	[0.76,0.97]
		U1 exposure|^d^	0.96	[0.88,0.99]	0.92	[0.77,0.97]	0.95	[0.85,0.98]
		Lower 1/3 Height|^d^	0.99	[0.96,0.99]	0.99	[0.97,1.00]	0.99	[0.98,1.00]
		Facial Height_n’|^d^	0.99	[0.97,1.00]	0.99	[0.96,1.00]	1.00	[0.98,1.00]
		Inner Canthic Diameter|^d^	0.87	[0.66,0.96]	0.83	[0.56,0.94]	0.40	[−0.12,0.75]
		g’-TVL|^d^	0.93	[0.80,0.98]	0.91	[0.75,0.97]	0.96	[0.89,0.99]
		pn’-TVL|^d^	0.96	[0.90,0.99]	0.97	[0.92,0.99]	0.91	[0.76,0.97]
		mp’-TVL|^d^	0.94	[0.83,0.98]	0.88	[0.68,0.96]	0.89	[0.71,0.96]
		li’-TVL|^d^	1.00	[1.00,1.00]	1.00	[0.99,1.00]	1.00	[0.99,1.00]
		sm’-TVL|^d^	1.00	[0.99,1.00]	1.00	[0.99,1.00]	0.99	[0.96,1.00]
		pg’-TVL|^d^	0.94	[0.83,0.98]	1.00	[1.00,1.00]	0.99	[0.98,1.00]
		gn’-TVL|^d^	0.97	[0.92,0.99]	1.00	[0.99,1.00]	0.99	[0.98,1.00]
Airway	Linear	H-MP|^d^	1.00	[0.99,1.00]	1.00	[0.99,1.00]	1.00	[1.00,1.00]
		H-C|^d^	0.99	[0.98,1.00]	1.00	[0.99,1.00]	0.99	[0.98,1.00]
	Areal	Airway, Mean|^a 3^	1.00	[1.00,1.00]	1.00	[1.00,1.00]	1.00	[1.00,1.00]
		Velopharynx, Mean|^a^	1.00	[0.99,1.00]	1.00	[1.00,1.00]	0.99	[0.98,1.00]
		Glossopharynx, Mean|^a^	1.00	[1.00,1.00]	1.00	[1.00,1.00]	1.00	[0.99,1.00]
		Airway, Min|^a^	1.00	[1.00,1.00]	1.00	[1.00,1.00]	1.00	[1.00,1.00]
	Voluminal	Airway|^v 4^	1.00	[0.99,1.00]	1.00	[0.99,1.00]	1.00	[1.00,1.00]
		Velopharynx|^v^	1.00	[0.99,1.00]	1.00	[0.99,1.00]	0.99	[0.96,1.00]
		Glossopharynx|^v^	0.97	[0.91,0.99]	0.98	[0.93,0.99]	0.92	[0.77,0.97]

^1^|° represents that the parameter is angular. ^2^|^d^ represents that the parameter is linear. ^3^|^a^ represents that the parameter is areal. ^4^|^v^ represents that the parameter is voluminal.

## Data Availability

The data presented in this study are available on reasonable request from the corresponding author. The data are not publicly available due to privacy issues and regulation policies in hospitals. All requests about the semi-automatic system, labeling, comparison and evaluation can be sent to the first author (K.Y.).
